# Dataset of FD&C Certified Food Dyes in Foods Commonly Consumed by Children

**DOI:** 10.1016/j.dib.2022.108806

**Published:** 2022-12-07

**Authors:** Arlie L. Lehmkuhler, Mark D. Miller, Asa Bradman, Rosemary Castorina, Mary-Ann Chen, Tonya Xie, Alyson E. Mitchell

**Affiliations:** a Department of Food Science and Technology, University of California-Davis, 1 Shields Ave, Davis, CA 95616, USA; b Office of Environmental Health Hazard Assessment, California Environmental Protection Agency, Sacramento, CA, USA; c Center for Environmental Research and Children's Health (CERCH), School of Public Health, University of California, Berkeley, CA, USA

**Keywords:** FD&C dyes, Food samples, Estimated dietary intake, High performance liquid chromatography

## Abstract

This is dataset describing the levels of Food, Drug, & Cosmetic (FD&C) dye in juice drinks, breakfast cereals, frozen desserts, ice cream cones, fruit flavored soft drinks, frostings & icings, fruit snacks/candy, decoration chips for baking, water enhancers, and flavored fruit drink powder. Data values are organized by absolute values, averages, SDs and % RSD. High performance liquid chromatography with a photometric diode array detector (HPLC-PDA) was used to measure dye levels and generate the data. These values can be used to calculate levels of dyes consumed within various populations, such as children, and compare them to accepted daily intake (ADIs) values established by the United States Food & Drug Administration (US FDA). The data are interpreted in “Survey of Certified Food Dye Levels in Food Samples Consumed by Children for Updated Exposure Levels” in the Journal of Food Additives and Contaminants: Part B.^1^

**Specifications Table**

**Table:** 

Subject	Analytical Chemistry
Specific subject area	Toxicology, Public Health
Type of data	Tables of absolute values, averages, SDs, % RSD and ranges of Food, Drug, & Cosmetic (FD&C) dyes in food samples across 10 categories.
How data were acquired	Dye levels were quantified on an Agilent 1200 HPLC system coupled with a photodiode array detector (Agilent Technologies. Memphis, TN) and equipped with an InfinityLab Poroshell 120 EC-C_18_ column (250 mm × 4.6 mm, i.d. with 4 μm particle diameter, Agilent Technologies, Memphis, TN). Software: ChemStation Rev. B.04.03
Data Format	Raw Data Analyzed
Parameters for data collection	Two to four brands of breakfast cereals, juice drinks, fruit flavored soft drinks, frostings/icings, decoration chips for baking, fruit flavored powder drinks, water enhancers, fruit snacks/candy, frozen desserts, and ice cream cones were collected in California from various supermarkets and sampled. Three different lots with differing identification numbers within each brand were selected for sample replication.
Description of data collection	A composite sample was generated for each lot in each brand of food. Each composite was diluted in water and sonicated until homogenous. The sample was centrifuged before concentration and loaded on one solid phase extraction (SPE) column, where it was washed and eluted. The final sample collected was dried using a Speed Vac then reconstituted in water for quantification on HPLC-PDA.
Data source location	University of California-Davis, Davis, California
Data accessibility	Repository Name: Mendeley Data Data identification number: DOI: 10.17632/7rpwxrphws.1 Direct URL to data: http://dx.doi.org/10.17632/7rpwxrphws.1
Related research article	Lehmkuhler, M. Miller, A. Bradman, R. Castroina, M. Chen, T. Xie, A. Mitchell, Levels of FD&C Certified Food Dyes in Food Commonly Consumed by Children. J. Food Composition and Analysis, 112, 2022, 104649. https://doi.org/10.1016/j.jfca.2022.104649

## Value of the Data

• These datasets are valuable for understanding levels of certified food dye present in the marketplace, estimating dietary dye intake and for monitoring trends in the use of dyes in food products.
• Those working in public health and allied fields can benefit from this data to understand variability of food dyes in food products.
• These data can further be applied to average heights, average weights, and dosing to determine the contribution of current levels of certified food dyes in these food categories to intake in children.

## 1 Data Description

Food, Drug, & Cosmetic (FD&C) dyes can be found in various food products that are commonly consumed by children and marketed toward children. Amounts of FD&C dyes in commercial products are proprietary and must be directly assessed to determine the level of dye intake attributed to these products. Herein, FD&C dyes were quantified in ten common food categories: breakfast cereals, juice drinks, fruit flavored soft drinks, water enhancers, fruit flavored powder drink, frostings/icings, decoration chips for baking, fruit snacks/candy, frozen desserts, and ice cream cones. The FD&C dyes measured include FD&C Yellow 5, Yellow 6, Red 40, Red 3, and Blue. Ten data files, organized by food category, are available in the data repository. For each food category, the data is presented in Tables.

Data in Brief Breakfast Cereals, Decoration Chips, Powdered Drinks, Frosting/Icing, Frozen Desserts, Fruit Snacks/Candy, Ice Cream Cones includes the average dye content (mg-1 kg) and the range of dye content in the food (Table 1) and the serving size as indicated on each brand's label (Table 2). Raw data is provided in Table 3 and includes raw data values for each brand and lot, the average value of dye for each lot, and the average, SD, and % RSD of dye for each brand. Included in Table 3 are the peak areas, adjusted peak areas with internal standards, concentration based on the calibration curve, and the value after back calculating the concentration with consideration of the sample preparation process.

Data in Brief Juice Drinks, Soft Drinks, and Water Enhancers includes the average dye content by brand and color (mg-1 kg ± SD) (Table 1), average density (g^−1^ ml, kg^−1^ ml) of the product (Table 2), the average dye content (mg-1 kg ± SD) and the range of dye content in the food (Table 3), serving size (Table 4) and the raw data values for each brand and lot, the average value of dye for each lot, and the average, SD, and % RSD of dye for each brand (Table 5). Included in Table 5 are the peak areas, adjusted peak areas with internal standards, concentration based on the calibration curve, and the value after back calculating the concentration with consideration of the sample preparation process.

## 2 Experimental Design, Materials and Methods

### 2.1 Materials for Analysis

Analytical standards were purchased for FD&C Yellow No. 5, Yellow No. 6, Red No. 40, Red No. 3, Blue No. 1, and Amaranth (formerly FD&C Red No. 2) from Millipore Sigma (Missouri, USA). HPLC Grade ammonium acetate was acquired from Spectrum Chemicals (California, USA). Formic acid (High Purity Grade) and methanol (HPLC Grade) were obtained from VWR. Acetonitrile (HPLC Grade, > 99.9%) and hydroxide solution (ACS Reagent 28.0-30.0%) was purchased from Millipore Sigma (Missouri, USA). Waters Oasis WAX 3 cc Vac Cartridges (60 mg sorbent per cartridge, 60 μm particle size) were purchased from Neta Scientific (New Jersey, USA).

### 2.2 Samples for Analysis

Ten categories of foods were identified as being commonly consumed by children and were included for analysis herein. These categories include breakfast cereals, juice drinks, fruit flavored soft drinks, water enhancers, fruit flavored drink powders, frostings/icings, decoration chips for baking, fruit snacks/candy, frozen desserts, and ice cream cones Lehmkuhler et al. [1] Primary samples included three independent lots from 2-3 brands (depending upon availability) in each category (6 or 9 samples of each brand in each category). Products that were comprised of individual units of a different color (e.g. red, yellow or blue ice cream cones) were separated by color and evaluated, rather than a forming a composite of all colors. These products included juice drinks, fruit snacks/candy, and ice cream cones. Samples were extracted and evaluated in duplicate.

### 2.3 Standards

FD&C standard dyes and amaranth (internal standard) were combined in MilliQ water and diluted to established an eight-point calibration curve at concentrations over the range of 0.25, 0.5, 1, 2.5, 5, 10, 25, 50 mg L^−1^ for quantification.

### 2.4 Sample Preparation

The breakfast cereals, ice cream cones, frozen desserts, and decoration chips for baking were homogenized by prior to dye extraction. Frozen desserts were thawed and stirred prior to extraction. Samples were prepared by putting either 500 mg (frostings/icings and flavored fruit powder drink mix), 1 g (ice cream cones, breakfast cereals, frostings/icings, and decoration chips) or 3 g (juice drinks, frozen desserts, and soft drinks) sample was weighed into a 50 mL conical tube, and Milli-Q water was added to a final volume of 30 mL. Samples were sonicated for 30 min at 55 ± 5°C with intermittent vortexing every 10 mins during sonification. Samples were next centrifuged at 5000 RPM for 10 mins and the supernatant decanted. The supernatant then underwent a clean-up step using an Oasis WAX SPE cartridge (3 cc, 60 mg x 60 μm). Briefly, the SPE cartridge was preconditioned with 1 mL of methanol, followed by 1 mL of water. Next, a 3 mL aliquot of sample was loaded onto the preconditioned column using gravity flow. The cartridge then was washed with 1 mL of 2% formic acid in water (v/v), followed by 1 mL of methanol. Dyes were eluted with 2 mL of 5% ammonia in methanol (v/v) into glass tubes. The samples were centrifuged until they were dry using a SpeedVac (Savant SpeedVac, ThermoFisher, Massachusetts, USA). A 10 µL aliquot of 250 µg^−1^ ml amaranth in water was added to each sample as an internal standard. The samples were then brought up in 500 µL of MilliQ water, and filtered through a 0.2 um filter into a vial for injection on an HPLC-PDA instrument.

### 2.5 Instrument Conditions

HPLC analysis was prepared on an Agilent 1200 HPLC system coupled with a photodiode array (PDA) detector (Agilent Technologies, Memphis, TN) using an Agilent InfinityLab Poroshell 120 EC-C_18_ column (250 mm × 4.6 mm, i.d. with 4 μm particle diameter, Agilent Technologies, Memphis, TN). The mobile phase consisted of 10 mmol L^−1^ ammonium acetate in water for A and acetonitrile for B. A gradient elution of 0 min (3% B), 0−2 min (3% B), 2−5 min (10% B), 5−10 min (30% B), 10–12 min (33% B), 12−15 min (3% B), 15–17 min (3% B) at a flow rate of 1 mL min^−1^ was selected for optimum peak resolution and efficiency. The column temperature was maintained at 25°C with a 50 µL injection volume. Peak detection occurred at 420 nm, 480 nm, 520 nm, 609 nm, and 620 nm with the full scan PDA ran over 200–650 nm. Peak identification was confirmed by retention time and spectral characteristics of the standards.

## Declaration of Competing Interest

The authors declare that they have no known competing financial interests or personal relationships which have, or could be perceived to have, influenced the work reported in this article.

## Data Availability

Dataset of Certified Food Dye Levels in Food Samples Consumed by Children for Updated Exposure Levels (Original data) (Mendeley Data)

## Appendix Supplementary materials

Image, application 1
